# Dr. Benedict's Winter Retreat at Magnolia

**Published:** 1858-01

**Authors:** 


*Dr. Benedict's Winter Retreat at Magnolia.*—We would call the attention of physicians who have patients in delicate health, who are benefited by winters in the South, to the establishment of Dr. Benedict, at Magnolia, Florida. Dr. B. has himself suffered from pulmonary disease, but has recovered, and deems his improvement in a great measure, due to the influence of the salubrious and equable climate of Florida. By his circular, physicians will see that the retreat presents every facility for healthy exercise and amusement, while the climate is in every way suited to the invalid and the luxurious seeker after comfort. Access is easy, living cheap, and everything which can conduce to the comfort of the inmates is provided.

Dr. B. is well known as a practitioner in New York and Philadelphia, and is provided with excellent references. F.

